# Predictive Models for Compound Binding to Androgen and Estrogen Receptors Based on Counter-Propagation Artificial Neural Networks

**DOI:** 10.3390/toxics11060486

**Published:** 2023-05-26

**Authors:** Mark Stanojević, Marija Sollner Dolenc, Marjan Vračko

**Affiliations:** 1 BiSafe d.o.o., 1000 Ljubljana, Slovenia; mark.stanojevic@bisafe.si; 2 Faculty of Pharmacy, University of Ljubljana, 1000 Ljubljana, Slovenia; 3 National Institute of Chemistry, Hajdrihova 19, 1000 Ljubljana, Slovenia

**Keywords:** CPANN, androgen receptor, estrogen receptor, endocrine-disrupting chemicals

## Abstract

Endocrine-disrupting chemicals (EDCs) are exogenous substances that interfere with the normal function of the human endocrine system. These chemicals can affect specific nuclear receptors, such as androgen receptors (ARs) or estrogen receptors (ER) α and β, which play a crucial role in regulating complex physiological processes in humans. It is now more crucial than ever to identify EDCs and reduce exposure to them. For screening and prioritizing chemicals for further experimentation, the use of artificial neural networks (ANN), which allow the modeling of complicated, nonlinear relationships, is most appropriate. We developed six models that predict the binding of a compound to ARs, ERα, or ERβ as agonists or antagonists, using counter-propagation artificial neural networks (CPANN). Models were trained on a dataset of structurally diverse compounds, and activity data were obtained from the CompTox Chemicals Dashboard. Leave-one-out (LOO) tests were performed to validate the models. The results showed that the models had excellent performance with prediction accuracy ranging from 94% to 100%. Therefore, the models can predict the binding affinity of an unknown compound to the selected nuclear receptor based solely on its chemical structure. As such, they represent important alternatives for the safety prioritization of chemicals.

## 1. Introduction

Endocrine-disrupting chemicals (EDCs) are exogenous chemicals that interfere with the normal function of the human endocrine system through a variety of mechanisms and, as such, represent a global human health concern [1,2]. Certain chemicals can act through specific nuclear receptors, such as estrogen receptors (ERs) or androgen receptors (ARs), which are involved in the regulation of many complex physiological processes in humans [3]. All of the nuclear receptors mentioned (ARs, ERs) have an amino terminal group responsible for a ligand-independent activation of transcription, a central DNA-binding domain, and a ligand-binding domain at the carboxyl end. After binding to the ligand, the receptors translocate to the nucleus and bind to specific transcription elements to eventually trigger the transcription of the target genes [4]. It should also be noted that ARs and ERs can also act through very rapid non-genomic biological responses by triggering signaling circuits initiated outside the nucleus. This rapid action occurs through the interaction of nuclear receptors with various signal or scaffold molecules and has been shown to be involved in cell cycle control, proliferation, migration, and exclusion of steroid receptors from the cell nucleus [5,6,7].

Several physiological processes are regulated by estrogens through two estrogen receptors, ERα and ERβ. ERα is mainly expressed in the uterus, ovaries, breast, liver, kidney, bone, and white adipose tissue, whereas ERβ expression is found in the ovaries, male reproductive organs, prostate, central nervous system, cardiovascular system, lung, colon, kidney, and immune system. ERs are found predominantly in the nucleus, but also in the cytoplasm and mitochondria [8]. ERs have long been studied for their role in controlling the expression of the genes involved in vital cellular processes, such as proliferation, apoptosis, and differentiation. ERα and ERβ have been shown to have opposing effects on cell proliferation. While ERα activation promotes cell proliferation, ERβ activation suppresses cell proliferation and is associated with apoptosis [9]. Given the pleiotropic functions of ERs, the dysregulation of their signaling pathways contributes to a variety of diseases, including hormone-dependent breast, endometrial, and ovarian cancers, as well as neurodegenerative diseases, cardiovascular diseases, and osteoporosis [5].

ARs play an important role in the development and maintenance of the reproductive, musculoskeletal, cardiovascular, immune, neural, and hematopoietic systems [10]. The most abundant androgen is testosterone, which is widely considered the male sex hormone. Other androgens, such as dihydrotestosterone (DHT) and androstenedione, are necessary for the growth of the male reproductive system [4]. Androgens are present in varying amounts in both males and females. Dysregulations of the androgen receptor have been associated with cancers such as prostate cancer, breast cancer, ovarian cancer, and pancreatic cancer, as well as diabetes mellitus and muscle atrophy [11,12,13,14,15].

Therefore, identifying EDCs and reducing further exposures are more important than ever. To achieve this, the OECD has issued the Conceptual Framework for Testing and Assessment of Endocrine Disruptors [16], which provides a five-step workflow. Although the document is not prescriptive, it offers suggestions for possible next steps in testing. Unfortunately, there are very few data from in vivo tests such as the uterotrophic and Hershberger tests. Although very valuable, because they reflect organ-level changes resulting from interactions of xenobiotics with the endocrine system in the physiological state, such in vivo assays are expensive, time-consuming, and require large numbers of animals. In vitro receptor binding assays for estrogen and androgen systems are more commonly used to evaluate the endocrine-disrupting potential of chemicals, but only a fraction of all chemicals has been tested. Therefore, most chemicals have little or no data on their endocrine-disrupting properties, so a Tier 1 approach based on non-testing information, such as in silico approaches, appears to be the best tool for screening and prioritizing chemicals for further testing.

Artificial neural networks (ANN) have been used effectively in many areas of life science [17,18,19,20]. ANN functions as a self-learning system and it is typically a “black box”. By learning a set of examples with the correct answers, it can automatically derive logical solution principles and create a mapping between input and output. The fundamental advantage of modeling with neural networks is that they allow modeling of complicated, nonlinear relationships without assumptions about the structure of the model. Additionally, when accuracy is evaluated using a test set from the working database, the performance of most model predictions appears to be quite close to experimental measurements [21]. The counter-propagation artificial neural networks’ (CPANN) models are commonly used for the prediction of biological endpoints based on the chemical structure [22,23,24,25].

The CPANN models for predicting AR- and ER-mediated endocrine disruption are discussed in the paper. The use of the CPANN mapping technique allows us to identify the distribution of chemicals based on their structural similarity across the self-organizing map (SOM) and to distinguish such related chemicals based on their activity.

## 2. Data

For modeling, we created a dataset of structurally heterogeneous compounds from published data, including compound name, CAS, QSAR-Ready SMILES (SMILES representations of desalted, de-isotoped, stereo-neutral forms of chemical structures associated with specific chemical substances), and activity details (available in the Supplementary File supdata.xlsx).

Activity data were taken from the CompTox Chemicals Dashboard (https://comptox.epa.gov/dashboard/, accessed 10 October 2022) [26]. This is a publicly available, web-based application developed by the U.S. Environmental Protection Agency to provide access to systematically compiled and consolidated chemistry, toxicity, and exposure information for more than 900,000 chemicals. All Tox21 data used are assays redouts obtained by measuring reporter genes via receptor activity and developed using an inducible reporter (β-lactamase induction) detected using the GAL4-β-lactamase reporter gene [27]. The shift in fluorescence emission (from green to blue color) is used to identify the activation of the reporter gene as a result of ligand GR binding to the glucocorticoid response element. The β-lactamase reporter gene assay has two readouts: background (Channel 1, ch1, or green channel) and gene expression (Channel 2, ch2, or blue channel), which are used to calculate a ratio (ch2/ch1) for analysis. Details of assay protocols are described in EPA publication Toxicity Forecaster (Toxcast) In Vitro Assays [28]. Potentially active compounds (positive hit calls) were then determined by curve fitting using the ToxCast Data Pipeline [29].

Six available assays were included in our study and are listed in Table 1.

Due to the large differences between the results of different laboratories, only compounds found to be positive in at least 50% of the laboratories. A similar number of compounds, identified as negative in the CompTox Chemicals Dashboard [26], was chosen. The compounds that were selected were those that were tested as active the least in individual laboratories.

## 3. Methods

The DRAGON [30] program package was used to calculate 3690 structural descriptors for all compounds. The descriptors that had negligible variance or were not calculated for all compounds were removed and then analyzed using the principal component analysis (PCA) method. PCA is a popular method for analyzing multidimensional data [24,25,31,32] and is a mathematical transformation of the original variables into new variables or principal components (PCs). The new variables are sorted according to the proportion of variance they contain. Typically, only a handful of new variables is necessary to describe the entire variance hidden in the data. In the reported case, thousands of descriptors were replaced with 22 new variables.

The CPANN method was used for modeling, which is an example of a self-organizing map technique developed for analyzing data in multidimensional space. This technique is based on a nonlinear projection from multidimensional space onto a network of neurons organized as a two-dimensional map. To achieve a topology-preserving projection, a nonlinear algorithm called training is used. A neuron is in fact a vector with a dimension equal to the number of descriptors. The weights are the individual components of the vectors determined by a nonlinear iterative training algorithm. The objects are presented to the networks in this procedure, and the weights are modified to be ‘similar’ to the descriptor values. The training is repeated until the changes in weights between two successive steps fall below a certain threshold. Since the basic property of the trained network is that similar objects are close to each other, it is expected that chemicals with similar structural features form clusters [23]. An additional output layer is added to the CPANN to the network associated with the property. In our case, the output layer is two-dimensional, with one dimension corresponding to binders and the other to non-binders. When a compound is presented to the model, it is placed in a group of ‘similar’ objects. The output layer is used to read the property. In our case, the prediction was given as a two-dimensional vector with elements expressing the specific class. The threshold for classifying a substance as a binder or non-binder was set to 0.5. After the leave-one-out (LOO) tests, the technical parameter of the model, namely the dimension of CPANN and number of epochs, was determined. All models were finally trained with 500 epochs.

In our study, we used four common evaluation metrics to assess and compare the performance of the model, namely specificity (*SP*) (Equation (1)), sensitivity (*SE*) (Equation (2)), accuracy (*ACC*) (Equation (3)), and the Matthew correlation coefficient (*MCC*) (Equation (4)). Calculations were based on true positive (*TP*), true negative (*TN*), false positive (*FP*), and false negative (*FN*) predictions. These were derived from the statistics of the model’s prediction results, i.e., the confusion matrix.

(1) $$SP=\frac{TN}{TN+FP}$$

(2) $$SE=\frac{TP}{TP+FN}$$

(3) $$ACC=\frac{TP+TN}{TP+FN+TN+FP}$$

(4) $$MCC=\frac{\left(TP\times TN\right)-\left(FP\times FN\right)}{\sqrt{\left(TP+FP\right)\times \left(TP+FN\right)\times \left(TN+FP\right)\times \left(TN+FN\right)}}$$

## 4. Results and Discussion

In order to create the most accurate model possible, the careful selection of experimental data is paramount. A comparative analysis of ED-relevant data from several sources, including the CERAPP [33], CoMPARA [34,35], and Tox21 projects, as well as the ChEMBL and PubChem databases, revealed poor agreement between experimental values from different sources [36]. For this reason, we used data from only one source, the Tox21 screening program, where the results were obtained using the same assay and under the same conditions. Unfortunately, even in this case, the reproducibility of the assay is low. The determined assay reproducibility is 80.6% for the AR agonist, 67.2% for the AR antagonist, 76.9% for the Erα agonist, and 76.8% for the Erα antagonist [37]. No reproducibility was found for the ERβ assays. On the other hand, the comparison of different in silico models has shown that the size of the dataset is not directly related to the performance of the model [36]. Therefore, to ensure the highest quality of input data, we only included compounds that were identified as positive by at least 50% of the laboratories in the model.

**Figure 1:**
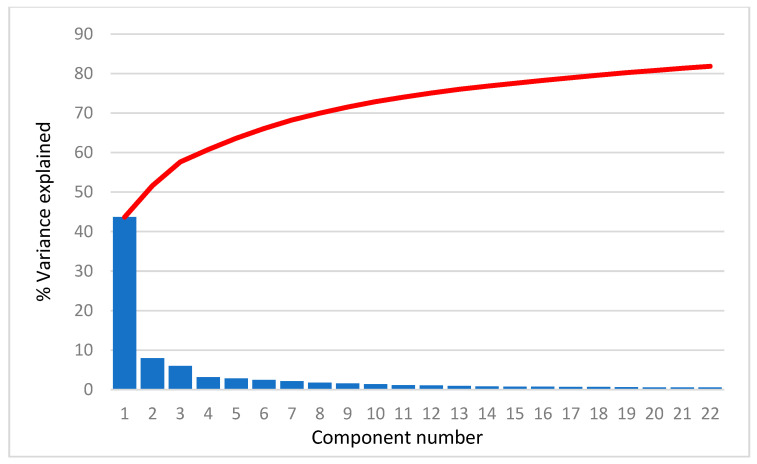
Scree plot for AR agonist. Blue bars represent the percentage of variance explained by each PC and red line represents sum of the variance explained by PCs.

For all selected compounds, 3690 structural descriptors were calculated using the program package DRAGON [30]. The descriptors that had negligible variance or were not calculated for all compounds were removed. In further PCA analysis, 2386, 2495, 2444, 2518, 2404, and 2484 descriptors were used for the AR agonist, AR antagonist, ERα agonist, ERα antagonist, ERβ agonist, and ERβ antagonist a, respectively. As an example, we present the scree plots (Figure 1) for AR agonists showing that the first PC carries more than 40% of the total variance, the second PC about 8.0–9.5% of the variance and the others even less. In fact, the scree plots are very similar for all cases studied. The common feature is that the first 22 PCs account for about 80% of the total variance.

**Figure 2:**
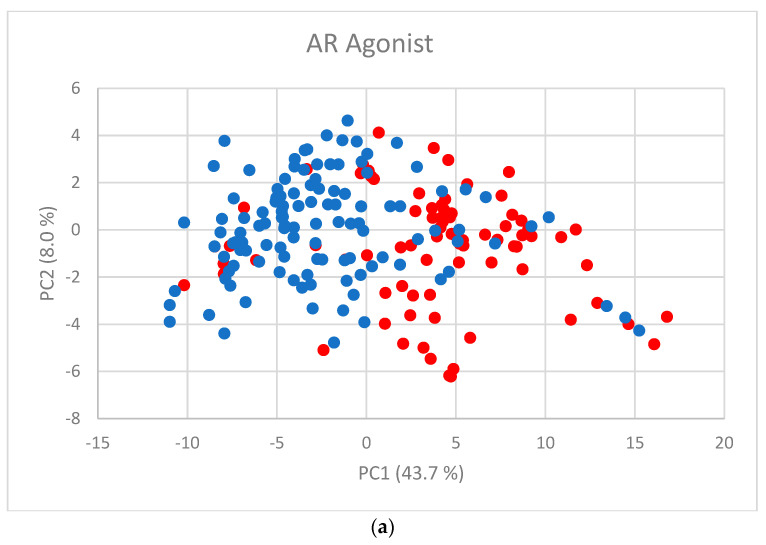

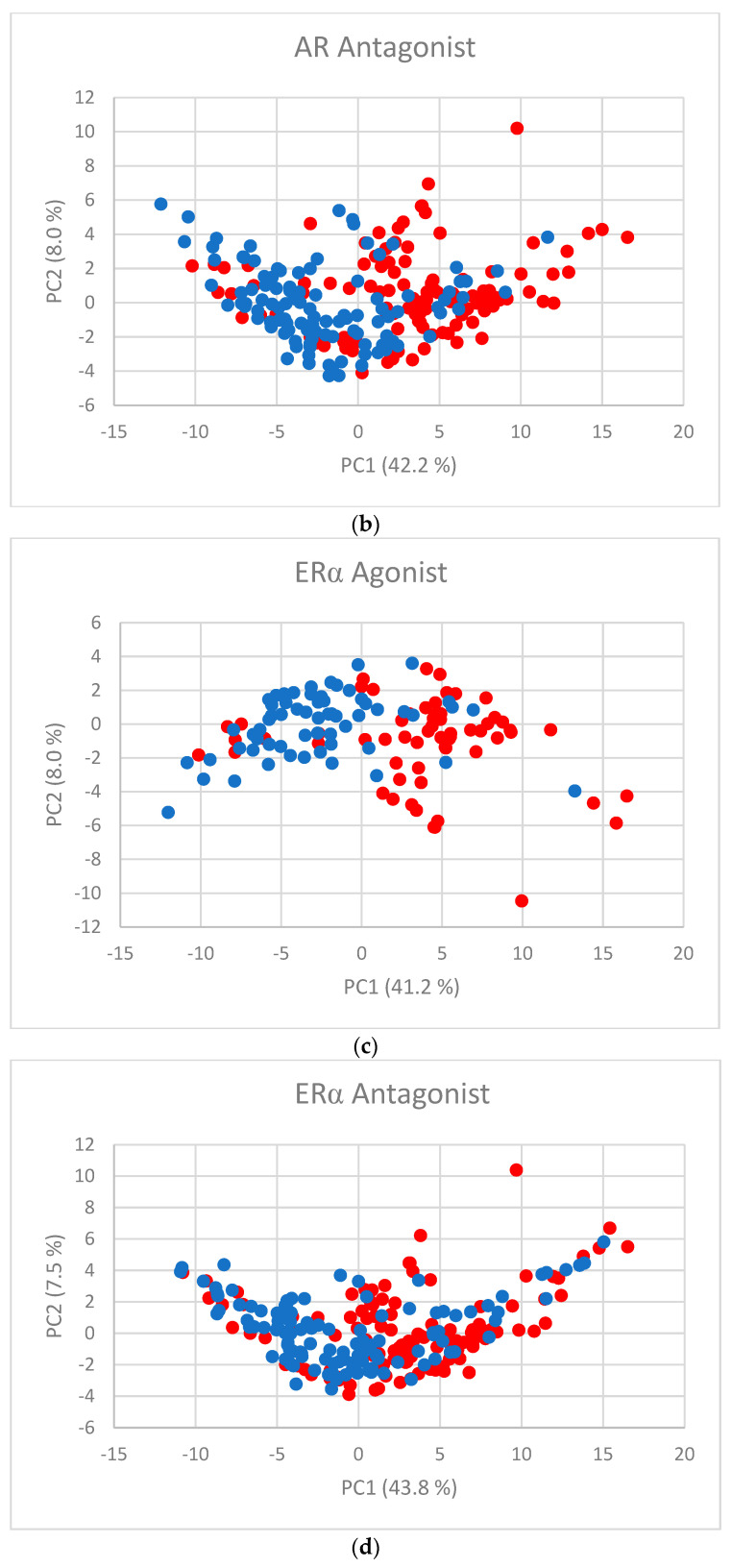

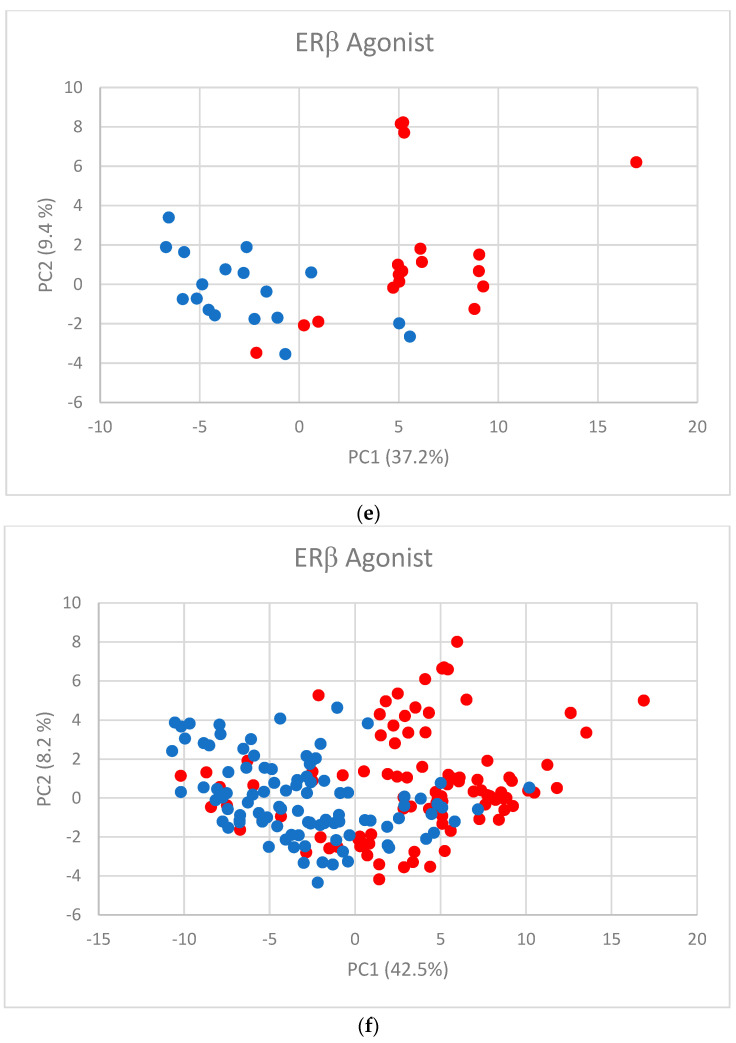
PC scores for (**a**) AR agonist, (**b**) AR antagonist, (**c**) ERα agonist, (**d**) ERα antagonist, (**e**) ERβ agonist, and (**f**) ERβ antagonist. Blue dots represent inactive substances and red dots represent active substances.

The first two PC scores (Figure 2a–f) show the separation between active and inactive compounds. Most of the inactive compounds are on the left of the line defined by the condition PC1 = 0, while the majority of active compounds are on the right of the line. The remaining PCs contribute little to the separation.

Subsequently, we considered the 22 PCs as input descriptors in the CPANN modeling. The architecture of the CPANN model and the LOO test are described in the above section. The LOO test results are shown in Table 2, considering different dimensions of CPANN.

**Figure 3:**
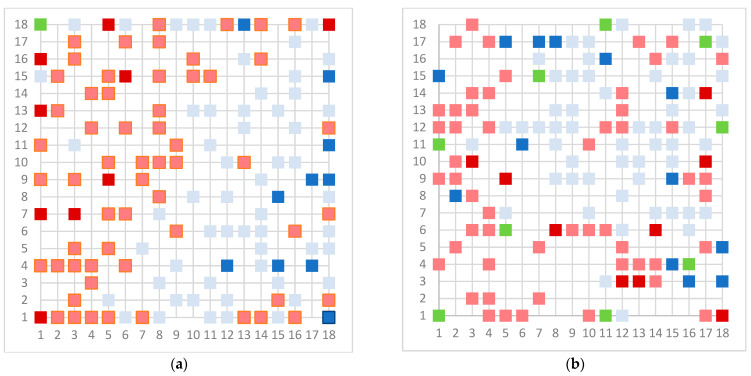

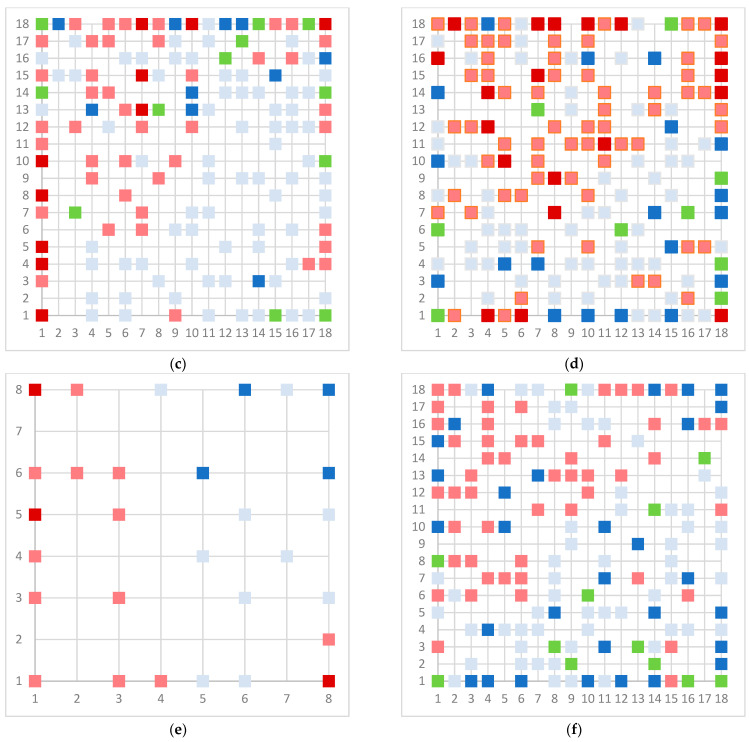
Top map of model for (**a**) AR agonist, (**b**) AR antagonist, (**c**) ERα agonist, (**d**) ERα antagonist, (**e**) ERβ agonist, and (**f**) ERβ antagonist. Light blue represents neuron with one inactive compound, dark blue neuron with two or more inactive compounds, light red represents neuron with one active compound, dark red represent neuron with two or more active compounds, and green represents conflict neuron, where at least one substance is classified differently than others.

As a final model, we chose the 18 × 18 dimension for the models, except for the Erβ-agonists 8 *×* 8 due to the small number of molecules. In the case of the Erβ-agonists, the model gives perfect separation, which is a consequence of the small number of molecules that have similar structures, especially for non-active molecules. The upper maps are shown in Figure 3.

The model for the AR agonist receptor shows near-perfect separation with only one misclassified molecule and an accuracy of 0.994. The only conflict (highlighted in green) in the AR agonist ‘recall-ability’ test presented in the top map is on the neuron (118) occupied by deslorelin and eledoisin, both short peptides with a molecular weight of >1000 g/mol and c-terminal pyrimidine, which are difficult to separate in the model. The top map of the AR antagonist shows nine conflict neurons. On neuron (1,6), there are four compounds (romidepsin, dronedarone, nelfinavir mesylate, and clindamycin palmitate) with molecular weights between 540 and 700 g/mol, which contain sulfur. Only clindamycin palmitate was non-binding in in vitro experiments, while the other three compounds were found to be binding. On the other hand, there are also four molecules on neuron (18, 9) presented in Table 3, all of which contain a heterocycle with nitrogen (1-butyl-4-methylpyridinium, 1-butylpyridinium, 1,8-diazabicyclo[5.4.0]undec-7-ene, and butylmethyl-imidazolium), with 1-butyl-4-methylpyridinium binding as an antagonist to AR in in vitro experiments, whereas the other three compounds were not binding. We would like to point out that 1-butyl-4-methylpyridinium and 1-butylpyridinium differ only by a methyl group attached to the pyridine, while the in vitro experiment shows a different classification.

Similarly, the compounds 1-butyl-4-methylpyridinium and 1-butyl-2-methylpyridinium at the conflict neuron (12, 6) in the ERα-antagonist model are structural isomers (Table 4). They have the same chemical formula but differ in the position of the methyl group. They were classified as non-binding and binding, respectively, in vitro, whereas our model classified both as non-binding.

In the ERβ-antagonist model, three structurally related compounds (fenticlor, bithionol, and phencapton) are found in the conflict neuron (1, 1) (Table 5). Although fenticlor was non- binding in the in vivo experiment, the other two were. All three molecules contain chlorine bound to an aromatic ring. However, fenticlor and bithionolate show greater similarity in structure, as both consist of two chlorinated phenols bridged with sulfur to form C1, differing only in the number of chlorines attached to the phenol. These examples clearly demonstrate the robustness of the model, as it only has difficulty classifying molecules that are structurally closely related.

Unfortunately, ED-relevant data from several sources showed poor agreement between experimental values from different sources [26]. Therefore, we were not able to create a test set for the external validation of the models. However, the LOO validation procedure performed results in a reliable and unbiased estimate of model performance.

Following a previous study in which a comparative analysis of docking models (Endocrine disruptome software and VirtualToxLab) and their interpretation using Tox21 in in vitro data was performed, our models show better accuracy than the selected docking models. The prediction accuracy of the endocrine disruptome model was 0.53 for AR, 0.58 for Erα, and 0.62 for Erβ, while Virtual ToxLab showed slightly better results of 0.57, 0.59, and 0.62, respectively [38]. In this regard, the QSAR models show better accuracy. The comparison of different published QSAR models for screening AR showed that the balanced accuracy ranged from 0.55 to 0.81 [26]. The QSAR model with the best performance was presented by Todorov et al. [39], and was based on the multiparameter formulation of the common reactivity pattern (COREPA) approach. Better results were obtained with OASIS, a QSAR platform covering both ER and AR binding [40]. Evaluation with ToxCast™ of in vitro binding data gave an accuracy of ER of 0.88 and of AR of 0.84 [41]. Despite the fact that the above QSAR models show excellent results in compound classification, we managed to further improve the accuracy (0.94–1) of the predictions (presented in Table 2) with CPANN. Moreover, with the exception of the endocrine disruptome, none of the aforementioned models distinguishes between agonists and antagonists, which is an additional added value of our models.

## 5. Conclusions

In the interest of avoiding animal testing, in silico modeling focuses on models for categorizing and grouping substances using various chemometric approaches. In this work, we present CPANN models for agonistic and antagonistic binding to the androgen and estrogen (ERα and ERβ) receptors. The structural properties of the compounds were fully described by the set of molecular structural descriptors given by DRAGON. Variable reduction was performed by the PCA-based clustering technique. In all cases, the first PC contributes about 40% to the total variance, and thus, plays a crucial role in the distribution of the objects. Score plots using the first two PCs show a weak separation between active and non-active compounds. Subsequently, we used CPANN models for classification. Notwithstanding the uncertainties in the classification of the compounds in the selected in vitro data, we divided the compounds into binders and non-binders. The models that were created were validated using statistical parameters for LOO, which showed that the models had a high level of balance and accuracy. Comparison with other QSAR and docking models presented in the literature with the CPANN models suggests that CPANN models are superior, although an absolute measure of comparison is not possible due to the different sets of compounds used. As far as we know, these are only QSAR models that distinguish between antagonists and agonists. This is an important detail that further increases their usefulness. Overall, the CPANN models we created are robust and predictive of new chemicals. Based on chemical structure alone, the models can predict the binding affinity of an unknown compound to the chosen nuclear receptor. Thus, these models provide a viable, effective, and practical tool for rapidly screening the endocrine activity of organic compounds and prioritizing them for further testing.

## Figures and Tables

**Table 1:** Assays readout used in the study.

Assay	Number of Substances in Database	Number of Substances Used for Modeling
TOX21_AR_BLA_AGONIST_RATIO	1009	156
TOX21_AR_BLA_ANTAGONIST_RATIO	2004	228
TOX21_ERA_BLA_AGONIST_RATIO	1398	123
TOX21_ERA_BLA_ANTAGONIST_RATIO	1617	231
TOX21_ERB_BLA_AGONIST_RATIO	1740	36
TOX21_ERB_BLA_ ANTAGONIST_RATIO	1966	194

Assays readouts were taken from the CompTox Chemicals Dashboard [26].

**Table 2:** LOO test results. Final models are presented in bold format.

	Androgen Receptor Agonist			Androgen Receptor Antagonist		
	16 × 16	**18 × 18**	20 × 20	16 × 16	**18 × 18**	20 × 20
TP	71	**77**	77	103	**105**	109
FP	0	**0**	0	2	**3**	2
TN	78	**78**	78	112	**111**	112
FN	7	**1**	1	11	**9**	5
Se	0.910	**0.987**	0.987	0.904	**0.921**	0.956
Sp	1.000	**1.000**	1.000	0.982	**0.974**	0.982
Acc	0.955	**0.994**	0.994	0.943	**0.947**	0.969
MCC	0.914	**0.987**	0.987	0.889	**0.896**	0.939
	**Estrogen receptor alfa agonist**			**Estrogen receptor alfa antagonist**		
	16 × 16	**18 × 18**	20 × 20	16 × 16	**18 × 18**	20 × 20
TP	58	**59**	60	104	**109**	106
FP	1	**1**	1	3	**2**	0
TN	60	**60**	60	112	**113**	115
FN	4	**3**	2	12	**7**	10
Se	0.935	**0.952**	0.968	0.897	**0.940**	0.914
Sp	0.984	**0.984**	0.984	0.974	**0.983**	1.000
Acc	0.959	**0.967**	0.976	0.935	**0.961**	0.957
MCC	0.920	**0.935**	0.951	0.873	**0.923**	0.917
	**Estrogen receptor beta agonist**			**Estrogen receptor beta antagonist**		
	6 × 6	**8 × 8**	10 × 10	16 × 16	**18 × 18**	20 × 20
TP	18	**18**	18	83	**89**	90
FP	0	**0**	0	3	**4**	2
TN	18	**18**	18	95	**94**	96
FN	0	**0**	0	13	**7**	6
Se	1	**1**	1	0.865	**0.927**	0.938
Sp	1	**1**	1	0.969	**0.959**	0.980
Acc	1	**1**	1	0.918	**0.943**	0.959
MCC	1	**1**	1	0.839	**0.887**	0.918

**Table 3:** Compounds on neuron (18, 9) in top map for AR antagonist.

Compound	Structure	AR Antagonist Classification	
		In Vitro	In Silico
1-Butyl-4-methylpyridinium	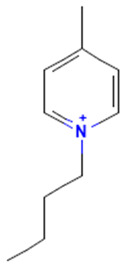	binding	non-binding
1-Butylpyridinium	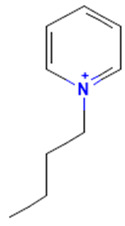	non-binding	non-binding
1-Butyl-3-methylimidazolium	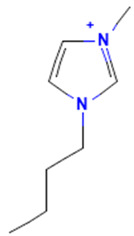	non-binding	non-binding
1,8-Diazabicyclo [5.4.0]undec-7-ene	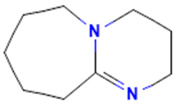	non-binding	non-binding

**Table 4:** Compounds on neuron (12, 6) in top map for ERα antagonist.

Compound	Structure	AR Antagonist Classification	
		In Vitro	In Silico
1-Butyl-4-methylpyridinium	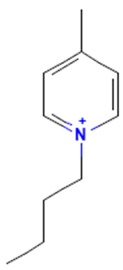	non-binding	non-binding
1-Butyl-2-methylpyridinium	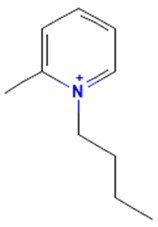	binding	non-binding

**Table 5:** Compounds on neuron (1, 1) in top map for ERβ antagonist.

Compound	Structure	ERβ Antagonist Classification	
		In Vitro	In Silico
Fenticlor	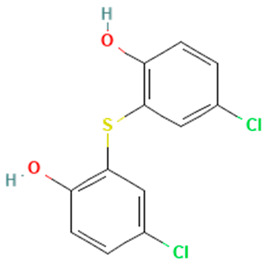	non-binding	binding
Bithionol	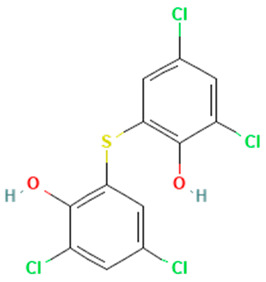	binding	binding
Phenkapton	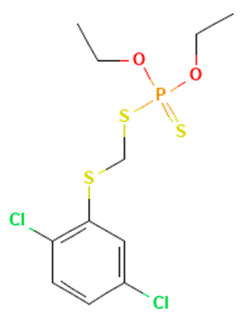	binding	binding

## Data Availability

In vitro activity data were taken from the CompTox Chemicals Dashboard: https://comptox.epa.gov/dashboard/ (accessed on 10 October 2022). Further data supporting the findings of this study are available from the corresponding authors upon reasonable request.

## Supplementary Materials

The following supporting information can be downloaded at: https://www.mdpi.com/article/10.3390/toxics11060486/s1, dataset including compound name, CAS, QSAR-Ready SMILES and activity details available in the Supplementary File supdata.xlsx.
